# Evolution of pandemic cholera at its global source

**DOI:** 10.1038/s41586-026-10340-x

**Published:** 2026-04-01

**Authors:** Amber Barton, Mokibul Hassan Afrad, Alyce Taylor-Brown, Nisha Singh, Chetan Thakur, Taufiqul Islam, Sadia Isfat Ara Rahman, Marjahan Akhtar, Yasmin Ara Begum, Taufiqur Rahman Bhuiyan, Ashraful Islam Khan, Neelam Taneja, Nicholas R. Thomson, Firdausi Qadri

**Affiliations:** 1 Wellcome Sanger Institute, Hinxton, UK; 2 Infectious Diseases Division, International Centre for Diarrhoeal Disease Research, Bangladesh, Dhaka, Bangladesh; 3 Department of Medical Microbiology, Postgraduate Institute of Medical Education and Research, Chandigarh, India; 4 Department of Infection Biology, Faculty of Infectious and Tropical Diseases, London School of Hygiene & Tropical Medicine, London, UK

**Keywords:** Bacterial genomics, Bacterial genetics, Phylogenetics

## Abstract

The seventh pandemic of cholera, caused by the seventh pandemic El Tor lineage of *Vibrio cholerae*, was previously shown to have emanated in three global waves from the Bay of Bengal, bordering Bangladesh and India^1^. However, the respective roles of the Ganges Delta and Basin regions in seeding these global pandemic waves were not known. Here we show that, although transmission events occur between Bangladesh and India, *V. cholerae* in the two countries has largely evolved separately over the past 20 years, apparently constrained by national borders rather than by hydrological features, such as the Ganges Delta and Basin. Evolution within Bangladesh was distinct from that seen in India, involving rapid gain and loss of genes and mobile genetic elements, particularly those involved in phage defence. The loss of these systems was associated with increased risk of severe disease and transmission outside Bangladesh. Lineage replacement in Bangladesh in 2018, resulting in a major change in phage defence systems, was accompanied by a rapid change in the lineage and anti-defence system of lytic phage ICP1. Here we show that the Ganges Basin, falling across Bangladesh and Northern India, rather than the Ganges Delta, probably acts as a global launch pad for pandemic disease. This shifts our understanding of Bangladesh as the purported global source of cholera and underscores the potential role of phage in controlling spread of lineages within the current seventh pandemic.

## Abstract (web summary)

The seventh cholera pandemic, driven by El Tor *Vibrio cholerae*, evolved separately in Bangladesh and India, with the Ganges Basin acting as the global source, and its evolution has been shaped by phage defence system changes, influencing severity and transmission.

## Main

Cholera, an acute diarrhoeal infection caused by *V. cholerae*, has caused seven global pandemics, of which the first six were caused by the classical biotype and thought to disseminate globally from the Ganges Delta^2^. At the beginning of the last century, the El Tor biotype, named after the El Tor quarantine station in Egypt where it was first observed, began to replace the classical biotype in epidemic disease. The seventh cholera pandemic, which started in Indonesia in 1960, is the longest-running pandemic for any pathogen^3^ with the largest recorded epidemics occurring in recent times, including Haiti in 2010 (ref. ^4^) and Yemen in 2016 (ref. ^5^) and 2019 (ref. ^6^). Since 2022, there has been an uptick in outbreaks. Countries such as Lebanon^7^ and Syria^8^ have reported cholera for the first time. There have been unseasonal outbreaks in Malawi^9^, and Bangladesh saw its largest outbreak ever in 2022 (ref. ^10^).

The seventh pandemic can be attributed to a single discrete genetic lineage named seventh pandemic El Tor lineage (7PET)^11^. Although 7PET phenotypically shares the same serogroup as the classical biotype (O1), it emerged independently from a non-epidemic El Tor biotype ancestor following acquisition of the CTXφ phage and two pathogenicity islands (*Vibrio* seventh pandemic islands (VSP)-I and VSP-II)^12^. Since 1960, 7PET has disseminated globally in three overlapping waves originating from the Ganges Basin^1^.

Global dissemination of 7PET appears as repeated clonal expansions, with 11 observed introduction events into Africa (termed T1 and T3–T12)^13^, three into Latin America (LAT1–LAT3)^14^ and eight into Europe (EUR1–EUR8)^15^. Although the dynamics of *V. cholerae* in specific outbreaks or restricted geographic sites have been explored^6,7,13^, the overall evolutionary dynamic has not been studied longitudinally across the ‘global home for cholera’ in the Ganges Basin, spanning India and Bangladesh. What is known is that two 7PET lineages have been consistently observed in India and Bangladesh over the past 15 years, previously named ‘BD1’ and ‘BD2’ (refs. ^10,16–20^). Although their genomes differ by fewer than 150 single-nucleotide polymorphisms (SNPs), they show marked variation in mobile genetic elements (MGEs), conferring differences in phage defences, antibiotic resistance and virulence. These include genomic islands (such as CTXφ, VSP-II and the superintegron^21^), SXT integrative and conjugative elements (SXT-ICEs) (such as ICE^TET^ and ICE^GEN^ (ref. ^22^)), plasmids (such as pCNRVC190243; ref. ^6^) and phage-inducible chromosomal island-like elements (PLEs)^23^. Although the role that many of these genetic elements play in disease severity is unknown, it has recently emerged in metagenomic analyses that the presence of *V. cholerae*-specific bacteriophage ICP1 attenuates disease^24^.

We collected and sequenced isolates from across Bangladesh (*n* = 1,516; 2014–2023) and North India (*n* = 794; 2002–2023) to generate, to our knowledge, the most comprehensive longitudinal dataset of cholera in the Ganges Basin so far. For many years, the primary global source of cholera was considered to be the brackish waters of the Ganges Delta and the Bay of Bengal. However, by tracking *V. cholerae* in the Ganges Basin, we observed that cholera transmission is constrained by national boundaries, and thus probably by population mobility, rather than reflecting the patterns we would expect if clinical disease was linked to a primarily environmental transmission path. We infer that, despite the high prevalence in Bangladesh, global dissemination of cholera is probably mediated by exportation from India. Within Bangladesh, *V. cholerae* shows unique patterns of evolution, particularly the rapid loss/gain of anti-phage elements, which are linked to increased risk of rice-water stool or severe dehydration. Furthermore, transmission of lineages outside Bangladesh to other countries may be compromised by the presence of these genetic elements.

## Rapid gene and MGE flux in Bangladesh

We sequenced isolates collected during a 2014–2018 nationwide systematic cholera surveillance study from sites across Bangladesh (*n* = 1,453) and those collected during an all-enteric disease screening campaign at the International Centre for Diarrhoeal Disease Research, Bangladesh (icddr,b) Hospital in Dhaka (*n* = 63), in which every 50th patient visiting was enrolled and tested for enteric pathogens, resulting in 1,477 high-quality genomes. From India, we sequenced 794 genomes spanning eight Northern Indian states collected through referral, clinical and surveillance services provided by the Postgraduate Institute of Medical Education and Research (PGIMER), Chandigarh. Once placed in the context of 3,112 published global genomes (Supplementary Data 1 and Supplementary Fig. 1), we found that our isolates from Bangladesh and India predominantly fell within two phylogenetic clades corresponding to the previously described lineages BD1 and BD2 (ref. ^16^). With more data from the Ganges Basin and Delta, it is clear that the previously named BD1 clade encompasses not only the previously defined BD1 lineage but also transmission lineages T11–13 and LAT3. We refer to this lineage as supra-BD1 (sBD1). Within sBD1 and BD2, pairs of samples were separated by medians of 37 and 19 SNPs, respectively, whereas between sBD1 and BD2, samples were separated by a median of 112 SNPs. To provide the resolution needed to track *V. cholerae* across the Ganges Basin and Delta, we subdivided sBD1 and BD2 into discrete sublineages on the basis of an SNP distance threshold of 20 and numbered according to their placement within the global phylogeny (Supplementary Fig. 2).

**Fig. 1:**
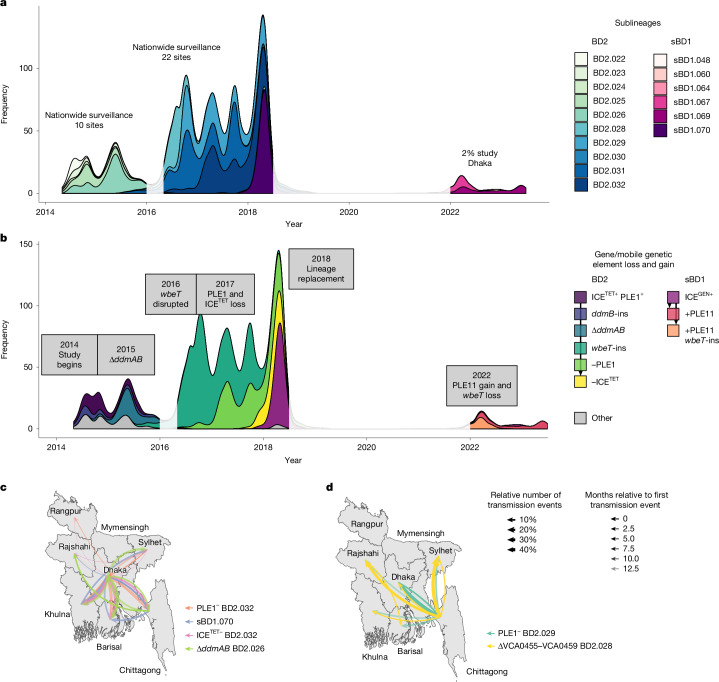
Dynamics of *V. cholerae* sublineages in Bangladesh and their genetic profiles over time. **a**, Abundance of each sublineage in the 2014–2018 nationwide surveillance study and 2022–2023 2% study shown over time, coloured by sublineage (see key). Time periods during which neither the 2014–2018 surveillance study nor the 2% study was active are shaded white. **b**, Abundance of each gene/MGE profile in the 2014–2018 nationwide surveillance study and 2022–2023 2% study shown over time, coloured by profile (see key). Time periods during which neither the 2014–2018 surveillance study nor the 2% study was active are shaded white. **c**,**d**, Phylogeography of different *V. cholerae* subpopulations of interest that disseminated from Dhaka (**c**) and Chittagong (**d**). To avoid sampling bias, only samples in the 2014–2018 nationwide surveillance study were included. For each administrative division, the source of the first introduction of each subpopulation to this division is indicated by an arrow. Arrows are coloured by the subpopulation of interest (see key); their size denotes the relative number of transmission events, and opacity denotes the months relative to the first transmission event (see key). Base maps in** c** and **d** with division boundaries were obtained from the bangladesh R package.

**Extended Data Fig. 1:**
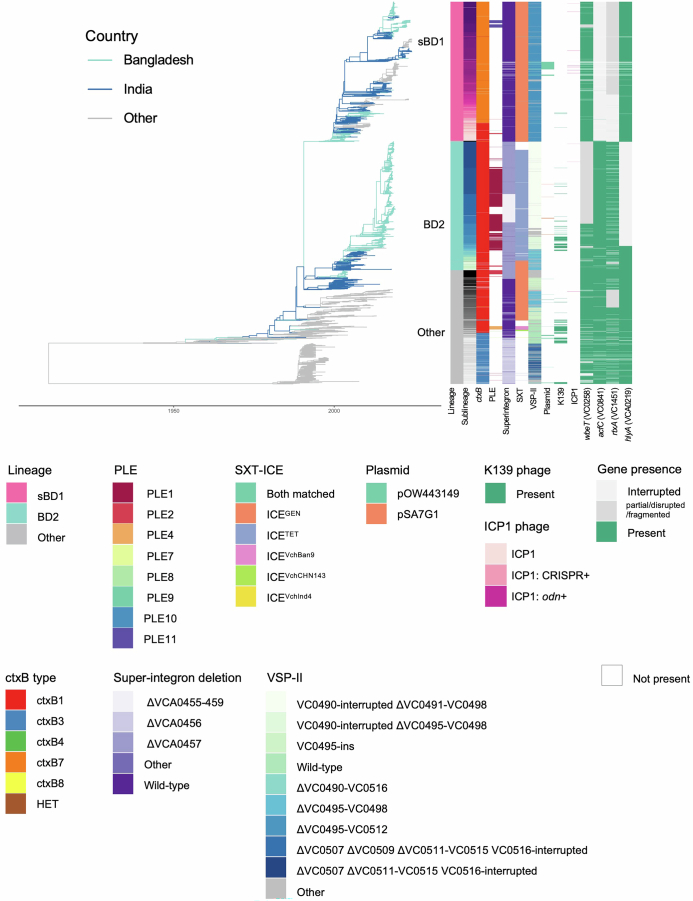
Time-scaled reference-mapped SNP phylogeny of 7PET samples. Outlier branches that did not follow a molecular clock, including the O139 lineage, have been excluded. Branches are coloured by whether their ancestral location was inferred to be India, Bangladesh or elsewhere. Previously defined sub-lineages and transmission events are indicated on the tree. Characteristics of each sample are indicated by the heatmap, including hierBAPS lineages and sub-lineages, *ctxB* allele, and presence of different mobile genetic elements and key genes.

**Extended Data Fig. 2:**
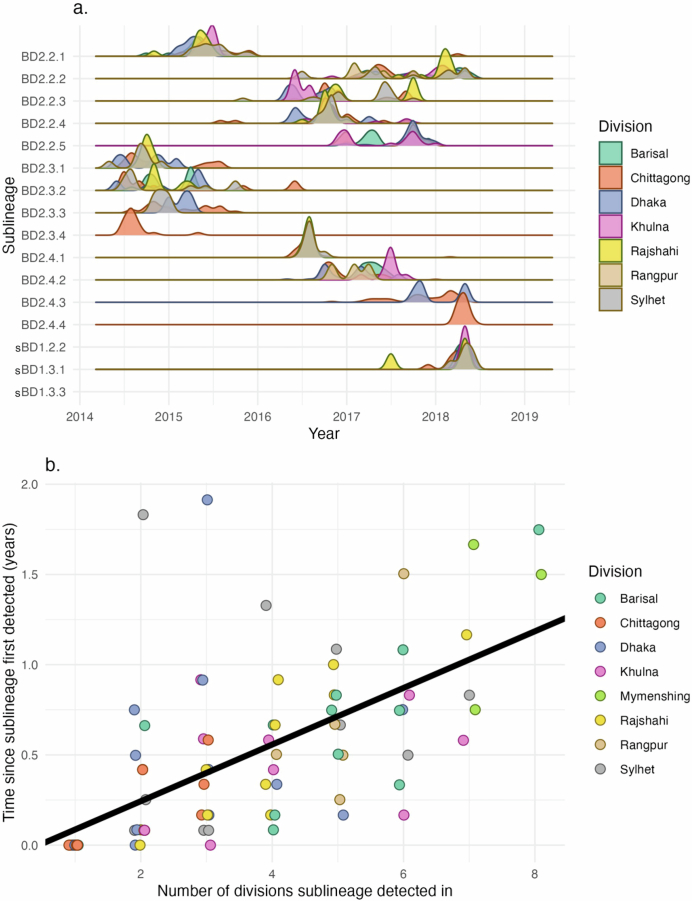
Dynamics of sub-lineages in each Bangladesh administrative division. a. Density plots of each sub-lineage coloured by division. b. Distribution of lag times between a sub-lineage being detected in a division, and being detected in the subsequent division. The line indicates a linear model used to calculate the length of time for sub-lineages to disseminate to all eight divisions.

From the two Bangladesh surveillance studies, BD2 predominated in Bangladesh from 2014 to 2017 (Fig. 1a) before being replaced in 2018 by sBD1, inferred by ancestral reconstruction to have been reintroduced from India (Extended Data Fig. 1). Notably, this is the only major introduction event observed for Bangladesh in this study; all other predominant sublineages circulating in Bangladesh were the same as or descended from sublineages from the previous year. The persistence of each sublineage varied (Fig. 1a), with an average duration of 16 months (interquartile range: 8–24 months) within the 2014–2018 systematic surveillance study. Of the 16 sublineages observed in Bangladesh during the surveillance study, 15 were detected in more than one administrative division. Considering pathogen migration rates, there was a lag of 12 months (linear model; 95% confidence intervals: 9–16 months) between first detection and a sublineage spreading to all eight divisions in Bangladesh (Extended Data Fig. 2). To infer the directionality of migration, we carried out repeated subsampling to account for variation in the sampling of acute watery diarrhoea cases by region. For each subsample, we constructed time-scaled phylogenies for sBD1 and BD2 and inferred the ancestral state of each node. Dhaka was the most common source of inferred interregional transmission events (49% events), followed by Chittagong (24% events; Supplementary Fig. 3).

**Extended Data Fig. 3:**
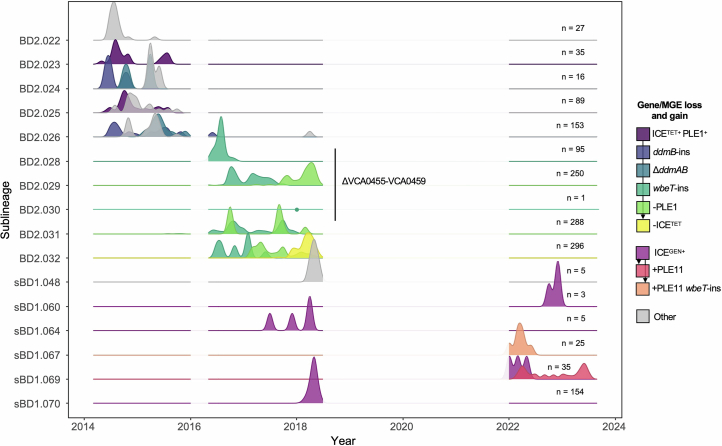
Density plots of each sub-lineage are coloured by gene and mobile genetic element profile (see key). For each sub-lineage the total number of samples are indicated on the right. Sub-lineages within the clade lacking VCA0455-VCA0459 are indicated by a label. A timeline at the top of the figure summarises the major changes that took place each year. Time periods where neither the 2014–2018 surveillance study nor the 2% study were active are shaded white.

**Extended Data Fig. 4:**
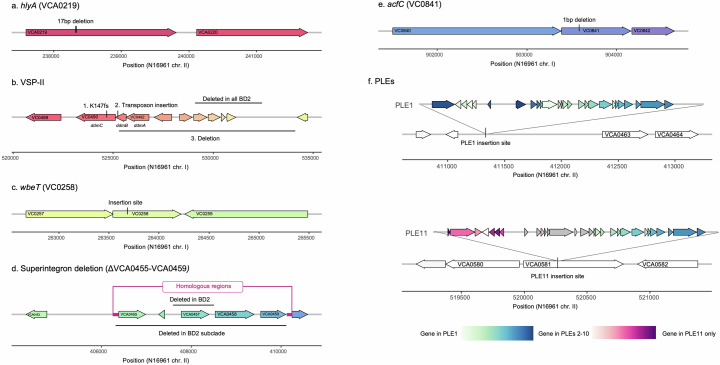
Genomic context of each of the major genetic changes undergone by sBD1 and BD2 in Bangladesh. a. *hlyA* (VCA0219) b. VSP-II region c. *wbeT* (VC0258) d. Deletion in the superintegron e. *acfC* (VC0841) f. PLEs. For PLE11, genes are coloured by whether they are shared with PLE1 (as determined by Panaroo v1.3.4 with a default sequence identity threshold of 0.98), rarer PLEs 2−10, or are unique to PLE11.

In addition to SNP variation in the core genome, *V. cholerae* genomes lost and gained individual genes and whole MGEs (Fig. 1b and Extended Data Fig. 3). By the first year of the surveillance study in 2014, the complete exotoxin *hlyA* gene (VCA0219) had been lost in 81% of BD2 genomes following a 17-base pair (bp) deletion and resultant frameshift mutation (Extended Data Fig. 4a). Furthermore, there was sequential degradation of *ddmABC*, an operon within VSP-II, which triggers cell suicide during phage infection or plasmid uptake^25^. This included a frameshift mutation in *ddmC*, which would probably render the entire *ddmABC* operon non-functional, followed by a transposase insertion in *ddmB* and then deletion of *ddmAB* and neighbouring genes *VC0493*–*VC0494* (Extended Data Fig. 4b). This was followed by disruption of *wbeT* (*VC0258*; Extended Data Fig. 4c), resulting in a phenotypic serotype switch in 2016, independent loss of anti-phage island PLE1 by two different sublineages in 2017 and loss of the SXT-ICE^TET^ element in 2017 (Fig. 1b). Furthermore, over the course of 2016, a monophyletic clade with deletion of *VCA0455*–*VCA0459* in the superintegron (Extended Data Fig. 4d), corresponding to sublineages BD2.028 and BD2.029, became more common, comprising 51% of the BD2 isolates sequenced in 2016 but falling to 27% in 2017–2018.

In 2018, the sBD1 sublineage sBD1.070 entirely replaced BD2 in Bangladesh. In contrast to BD2, sBD1.070 carried the SXT-ICE^GEN^ element, the *ctxB7* allele, a complete *hlyA* exotoxin gene, the entire *ddmABC* operon and intact *wbeT*. However, sBD1.070 carried a frameshift mutation in colonization factor *acfC* (*VC0841*; Extended Data Fig. 4e) owing to a nonsense mutation, and initially lacked a PLE anti-phage element. By 2022, when the 2% surveillance study in Dhaka began, three new sublineages had replaced sBD1.070, of which two (sBD1.067 and sBD1.069) descended from it and another was probably introduced from India (sBD1.060). The sBD1.060 sublineage was more closely related to the sublineages causing recent outbreaks in Pakistan, Lebanon and Malawi. In sBD1.067, the *wbeT* gene was disrupted by an insertion, conferring on it the Inaba serotype. Furthermore, both sBD1.067 and sBD1.069 had gained a new PLE island, here termed PLE11, in a different genomic position to PLE1 in BD2 (Extended Data Fig. 4f).

Next, to look for the temporal and spatial signatures in the loss and gain of gene functions in Bangladesh, we focused our analysis on genomes from the systematic 2014–2018 surveillance only to avoid sampling bias. Figure 1b and Extended Data Fig. 3 show that there are clear temporal patterns of loss: PLE1 and ICE^TET^ in sublineage BD2.032, PLE1 in BD2.029, *ddmAB* in BD2.026 and *VCA0455*–*VCA0459* in BD2.028. These changes were fixed in all derived sublineages (Supplementary Fig. 2). Using TreeTime, two patterns of geographical spread were evident: sBD1.3.1, PLE1^−^ and ICE^TET−^ BD2.2.2 and Δ*ddmAB* BD2.2.1 disseminated from Dhaka (Fig. 1c), whereas PLE1^−^ BD2.4.3 and Δ*VCA0455*–*VCA0459* BD2.4.1 disseminated from Chittagong (Fig. 1d).

**Extended Data Fig. 5:**
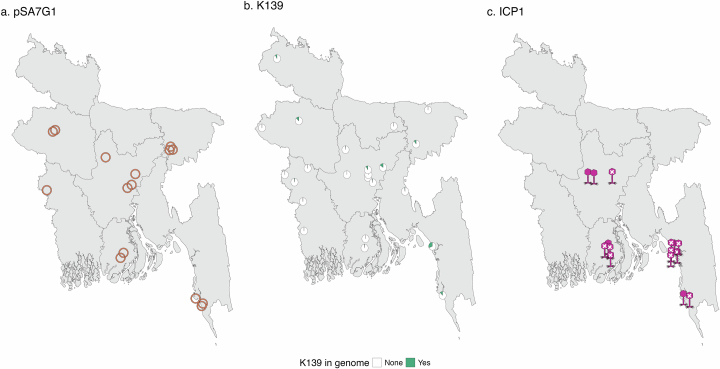
Geographic distribution of mobile genetic elements which did not become fixed in the population. a. Plasmid pSA7G1 b. Proportion of samples at each site with lysogenic K139 phage in the genome c. Location of *V. cholerae* samples co-sequenced with ICP1 lytic phage. Samples which were excluded from the main analysis due to lowered *V. cholerae* reads are indicated by a white cross. Base maps in **a**–**c** from Natural Earth (https://www.naturalearthdata.com/) under a Universal Public Domain CC0 licence.

In addition, sporadically acquired MGEs were observed, including plasmid pSA7G1 (Extended Data Fig. 5a), which was acquired several times in five BD2 sublineages distributed across six administrative divisions. The functional relevance of pSA7G1 is unclear. The K139 lysogenic phage, which binds to the *V. cholerae* O1 antigen and carries the gene *glo* linked to virulence in mouse models^26,27^, was found in ten BD2 sublineages across seven administrative divisions, most commonly in Chittagong (Extended Data Fig. 5b). Notably, the ICP1 lytic phage was detected and sequenced from 15 *V. cholerae* samples and, similar to K139, was most commonly from samples taken in Chittagong (Extended Data Fig. 5c).

## Global dissemination from South Asia

**Fig. 2:**
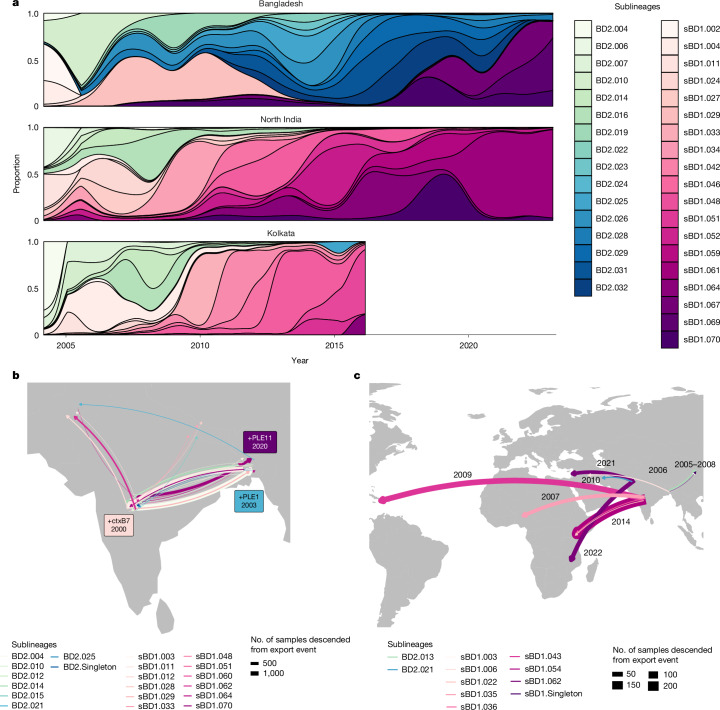
Dynamics of *V. cholerae* sublineages in the Ganges Basin and international transmission. **a**, Abundance of each sublineage within Bangladesh, North India and Kolkata over time, including both samples collected in this study and previously published contextual samples. Only sublineages with ten or more samples are shown. **b**, Inferred transmission events and key gene/MGE gain events within South Asia. Arrows are coloured by sublineage and sized according to the number of samples in our global collection that descended from this transmission event. **c**, Inferred transmission events from South Asia to other regions from 2003 to 2023. Arrows are coloured by sublineage and sized according to the number of samples in our global collection that descended from this transmission event, including not only the country indicated but also onward transmission to other countries. Base maps in **b** and** c** from Natural Earth (https://www.naturalearthdata.com/) under a Universal Public Domain CC0 licence.

**Extended Data Fig. 6:**
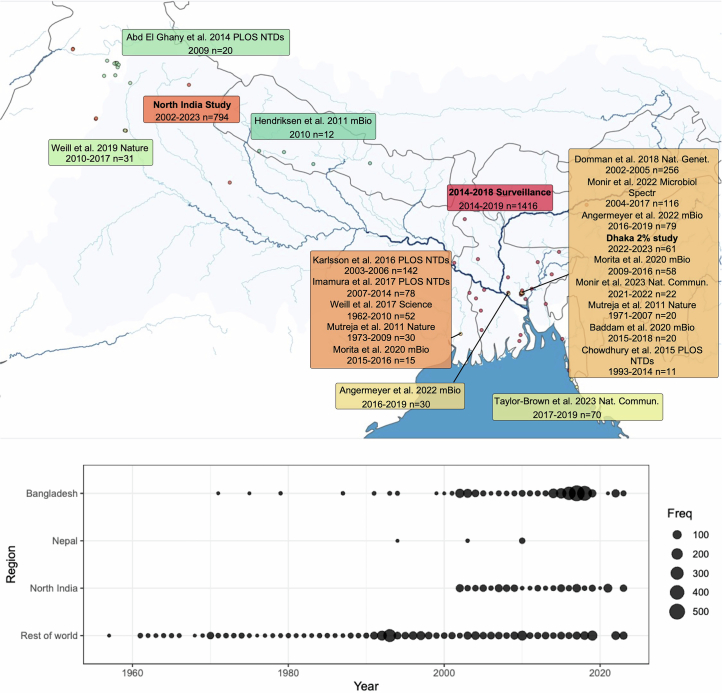
Studies and samples within and around the Ganges basin. Locations, numbers and collection years of samples collected for different studies within and around the Ganges basin. Base map from Natural Earth (https://www.naturalearthdata.com/) under a Universal Public Domain CC0 licence.

To understand the relationship between *V. cholerae* in Bangladesh and in other countries falling within the Ganges Basin, we analysed the 794 isolate genomes collected across North India (Extended Data Fig. 6). Although both Bangladesh and India harboured sublineages falling within sBD1 and BD2, the two countries followed distinct temporal patterns of sublineage replacement (Fig. 2a). Following co-circulation of both sBD1 and BD2 in Bangladesh and India from 2004 to 2011, sBD1 became predominant in India, representing 94% of samples by 2011, whereas BD2 became predominant in Bangladesh, representing 95% of samples by 2013. Of the 11 BD2 sublineages present in Bangladesh from 2013 to 2018, ten were contained within the national boundaries of Bangladesh and not found in any other country in the world, suggesting that during this time period BD2 was evolving in Bangladesh in isolation. In both countries, Tajima’s *D* consistently fell below −4, suggestive of selective sweeps, and nucleotide diversity varied over time, increasing during time periods when several 7PET lineages were present (Supplementary Fig. 4). The PLEs that typified the sublineage isolates circulating in Bangladesh were rare outside the country, as was loss of *ddmABC* and complete Inaba serotype replacement (Supplementary Fig. 5).

**Extended Data Fig. 7:**
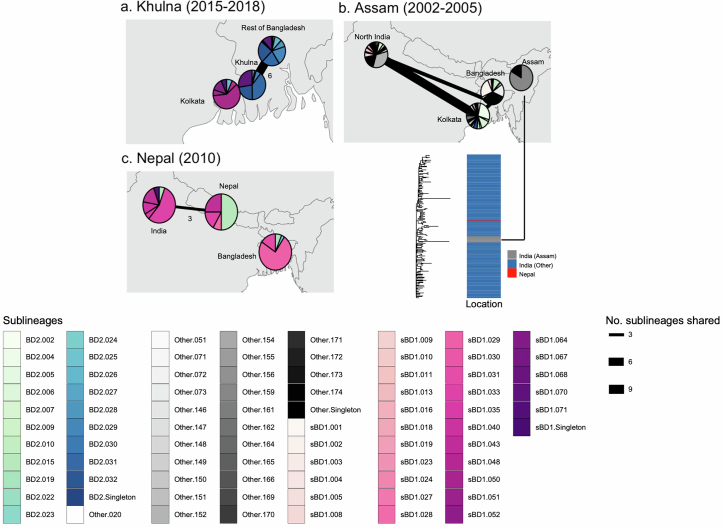
Sharing of sub-lineages between different locations within the Ganges basin. The proportion of each sub-lineage within a geographic location is represented by a pie chart. The width of segments joining each pie chart represent the number of sub-lineages shared between each location. a. Sub-lineages shared between Khulna, Kolkata and the rest of Bangladesh, from 2015–2018. b. Sub-lineages shared between Assam and North India, Kolkata and Bangladesh, from 2002–2005. A sub-tree displaying the phylogenetic context of samples from Assam is also shown. c. Sub-lineages shared between Nepal, India and Bangladesh in 2010.

Kolkata lies only 60 km from the Khulna division of Bangladesh and shares close proximity to the Sundarban wetlands, posited by Robert Koch in 1884 as being the probable source of cholera^28^. However, the temporal patterns of *V. cholerae* seen in Kolkata aligned with those in North India (Fig. 2a). Conversely, none of the 167 genomes taken in the Khulna division from 2015 to 2018 were in sublineages shared with Kolkata, and all 167 were in sublineages shared with the rest of Bangladesh (Extended Data Fig. 7a), suggesting transmission follows borders rather than hydrological features. The six samples collected in Assam (2002–2005) formed a phylogenetic cluster that was most closely related to other samples in India, despite its geographic proximity to Bangladesh (Extended Data Fig. 7b). Of the 12 samples collected in Nepal in 2010, 12 belonged to sublineages present in India at the time and none to sublineages present in Bangladesh (Extended Data Fig. 7c).

Next, to reconstruct the history of global transmission events from the time-scaled phylogeny of global 7PET (Extended Data Fig. 1), we inferred the ancestral state and date of each node (Supplementary Data 2). High transmission was evident between countries in South Asia but with more transmission events between Bangladesh and India (40 of 408) than any other pair of countries (Fig. 2b). Dissecting these events in more detail, we show that sBD1 and BD2 diverged separately from the T9 lineage in the late 1990s. In addition, sBD1 seems to have gained the *ctxB7* allele in India, subsequently spreading to Nigeria, to Kenya and to Haiti to cause the 2010 Haiti outbreak (Fig. 2c). Since then, most outbreak-causing global transmission events from South Asia seem to have been seeded from the pool of *ctxB7*^*+*^ sBD1 evolving in India (Fig. 2c). From 2003 to 2023, there were twice as many samples from Bangladesh as from India in our global phylogeny, yet 32.6 times as many samples outside South Asia were more recently descended from Indian transmission events (*n* = 359; 2003–2023) compared with Bangladeshi transmission events (*n* = 11; 2003–2023). To check whether this was attributable to differences in the intensity of sampling over time, we carried out repeated subsampling such that the numbers of samples from Bangladesh, India and the rest of the world were the same each year (Supplementary Fig. 6). Once again, we found that since 2000, a greater number of transmission events have originated from India.

Despite the high number of local transmission events between India and Bangladesh, 29 of 40 failed to give rise to more than ten descendant samples. In the 2010s, eight India-to-Bangladesh transmission events were evident from these data, of which seven failed to persist, resulting in fewer than ten patient samples in Bangladesh. Although BD2 became predominant in Bangladesh from 2013 to 2017, only one export event was observed from 2010 onwards, resulting in a single sample of BD2 being detected in Kolkata. Both BD2 and sBD1 gained PLEs after the introduction or reintroduction to Bangladesh. BD2 was introduced to Bangladesh around 2001 and gained PLE1 in Bangladesh 2 years later. Similarly, PLE11 was gained by sBD1 around 2020, 2 years after sBD1 rose to more than 50% of samples in Bangladesh. PLE11 was not detected in any country other than Bangladesh.

## Disease severity and phage specificity

Given the rapid dynamics of gene/MGE loss but comparatively low number of global transmission events originating directly from Bangladesh, we investigated how changes in genes and MGEs might affect fitness (infection potential, susceptibility to phage and antimicrobial sensitivity), as well as the potential of different lineages to move outside Bangladesh and the Ganges Delta.

**Fig. 3:**
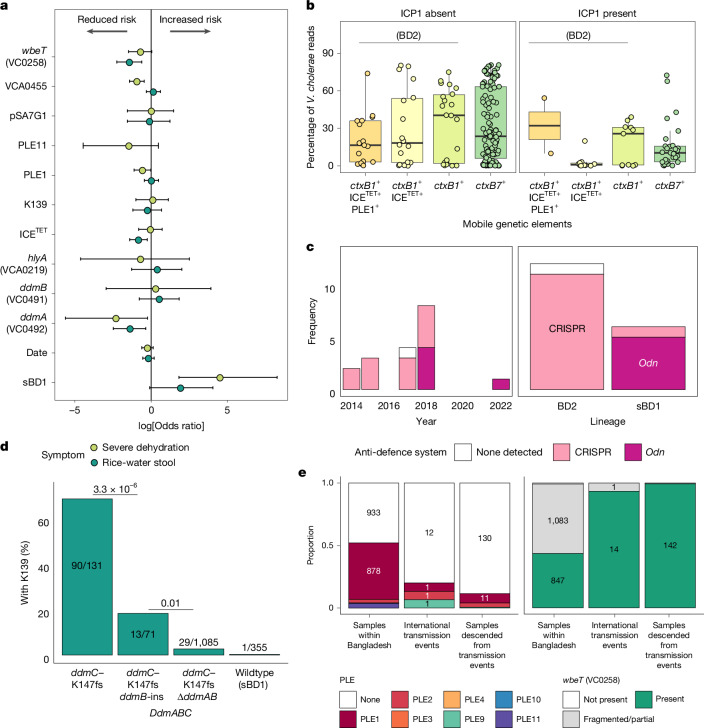
Relationships between *V. cholerae* genetic changes and phenotype. **a**, log-transformed odds ratios for the association of different genes and MGEs with rice-water stool and severe dehydration, adjusting for the presence or absence of other genes and MGEs, as well as the date and site of collection. The centre point indicates the calculated log-transformed odds ratio, and the error bars represent 95% confidence intervals; *n* = 1,617 biologically independent samples. **b**, Percentage of reads assigned to *V. cholerae* by Kraken in stool metagenomic data. Box plots indicate the median and interquartile range, with whiskers extending to the most extreme values within 1.5×  interquartile range. Samples were categorized by the presence of *ctxB7* and *ctxB1*, which, for this time period and location, delineated sBD1 and BD2; *ctxB1*^*+*^ samples were subcategorized on the basis of the detection of PLE1 and ICE^TET^; *n* = 230 biologically independent samples. **c**, Frequency of ICP1 anti-defence systems detected in samples from Bangladesh, by year and by lineage of the co-sequenced *V. cholerae*. **d**, Proportion of samples in which the lysogenic phage K139 was present in the genome, classified by which genes in the *ddmABC* antiviral system were present. *P* values are indicated for the results of a multivariate logistic regression testing the association (Wald test; two-sided) between changes in the *ddmABC* locus and K139, adjusted for the covariates indicated in Fig. 3a (ICP1, pSA7G1, SXT-ICE, PLE, *wbeT*, *VCA0455*–*VCA0459*, *hlyA*, lineage, date and site). No adjustment for multiple comparisons was applied. **e**, Representation of PLE^+^ and *wbeT*^*+*^ *V. cholerae* among the total number of samples from Bangladesh; inferred export events and the number of samples outside Bangladesh descended from these export events, from 2003 to 2023.

We first performed a multivariate logistic regression analysis to identify whether gene or MGE losses were associated with clinical severity (Fig. 3a), adjusting for the presence of other genes and MGEs. The presence of *ddmA* (*P* = 0.01), *wbeT* (*P* = 5.8 × 10^−4^) and ICE^TET^ (*P* = 0.005) was associated with a reduced risk of rice-water stool, whereas *VCA0455*–*VCA0459* (*P* = 0.0001) and PLE1 (*P* = 0.04) were associated with a reduced risk of severe dehydration, suggesting that their sequential loss may enhance disease severity. We considered that the loss of these genetic elements might allow faster replication and confer a selective advantage within the gut. We performed a systematic meta-analysis of 13,105 gut metagenomes from 21 cholera-endemic countries (Supplementary Table 1 and Supplementary Data 3) and found that, in the absence of ICP1, the proportion of reads in the stool metagenome occupied by *V. cholerae* increased from a median of 16.8% to 40.5% following the loss of ICE^TET^ by *V. cholerae* carrying *ctxB1*^*+*^ (and therefore probably lineage BD2; Fig. 3b). Even in the presence of ICP1, *V. cholerae* increased from 1.5% to 25.8% of the stool metagenome following ICE^TET^ loss.

**Extended Data Fig. 8:**
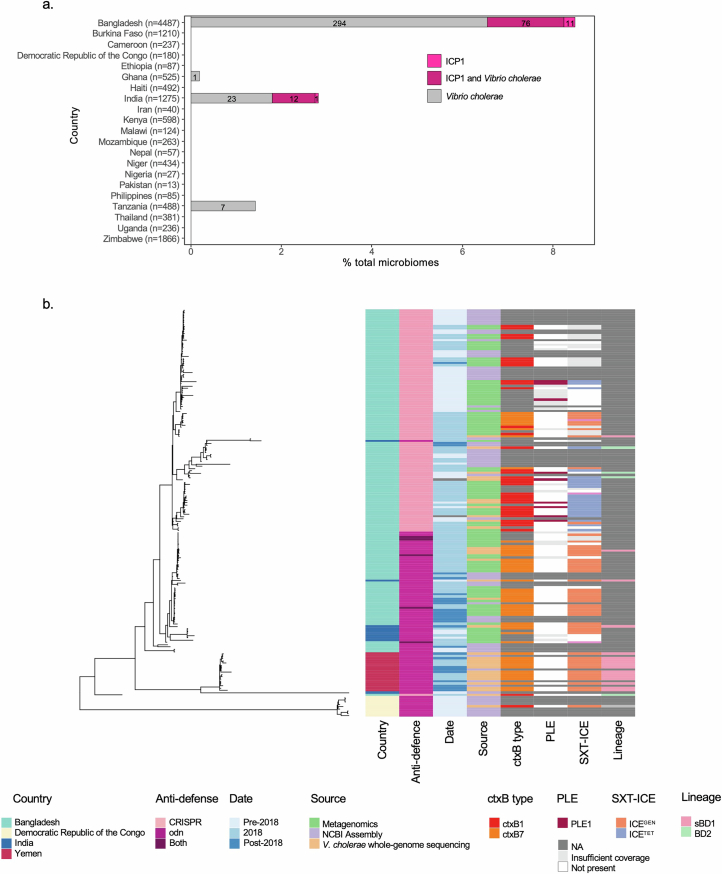
Global patterns of ICP1. a. Percentage of screened metagenomes from each country containing ICP1 (>0.01% reads using kraken2 v2.0.8) or *Vibrio cholerae* (presence/absence using Sylph v0.8.1). For each country, the total number of genomes screened is indicated in the y axis labels, and the absolute number of genomes containing ICP1 or *Vibrio cholerae* indicated by the bar chart labels. b. Mid-point rooted core-gene phylogeny of ICP1. Characteristics of each sample are indicated by the heatmap, including country, anti-defence system, source of the assembly, and characteristics of the co-sequenced *Vibrio cholerae* if present (PLE, SXT-ICE, and ctxB allele).

Our data showed a striking temporal link between ICP1 type and the circulating *V. cholerae* lineage. During the replacement of BD2 by sBD1, we observed a simultaneous switch in the ICP1 phage type co-sequenced alongside it, from ICP1 with a CRISPR–Cas anti-defence system (11 of 13 before April 2018) to ICP1 carrying the *odn* anti-defence gene (five of six from April 2018 onwards) (Fig. 3c). To better understand the global distribution and specificity of ICP1, the 13,105 gut metagenomes analysed above were screened for ICP1. We identified ICP1 in 87 metagenomes in Bangladesh and 13 in India, predominantly in metagenomes also containing *V. cholerae* (88%; Extended Data Fig. 8a). We generated 85 ICP1 genomes with more than 50% coverage from these metagenomes and integrated these with (1) publicly available ICP1 assemblies and (2) ICP1 co-sequenced with our global collection of *V. cholerae* genomes to build a global ICP1 phylogeny (Extended Data Fig. 8b and Supplementary Data 4). We found that ICP1 from Bangladesh, India, the Democratic Republic of Congo and Yemen formed distinct phylogenetic clusters. ICP1 with the CRISPR–Cas anti-defence system was phylogenetically distinct from *odn*^*+*^ ICP1, found only in Bangladesh and predominantly found alongside BD2. *Odn*^*+*^ ICP1 was identified in all four countries and seemed to be adapted to the sBD1 lineage. The *odn*^*+*^ ICP1 that emerged in Bangladesh in 2018 was more closely related to *odn*^*+*^ ICP1 in Kolkata in 2017 than those previously isolated in Bangladesh. We also identified genetic changes linked to the K139 phage susceptibility. Notably, *ddmABC* degradation was significantly associated with a decline in K139 prevalence (*P* = 3.3 × 10^−6^ and 0.01 for *ddmB* and *ddmA*, respectively; logistic regression adjusted for date, study site and presence or absence of other genes; Fig. 3d).

Given that PLE11 was not observed outside Bangladesh, we then examined whether PLEs in general compromise global transmission potential. Compared with the number of PLE^+^ genomes sampled in Bangladesh, PLEs were significantly under-represented among transmission events to other countries from 2003 to 2023 (Fig. 3e; *P* = 1.8 × 10^−4^; Fisher’s test) and even more under-represented within the genomes of *V. cholerae* descended from those events (*P* < 2.2 × 10^−16^; Fisher’s test). None (0 of 26) of the exported *V. cholerae* from India from 2003 to 2023 were PLE^+^. Similarly, the Inaba serotype *V. cholerae* lacking complete *wbeT* was rarely transmitted out of Bangladesh (*P* = 8.4 × 10^−5^; Fisher’s test).

**Extended Data Fig. 9:**
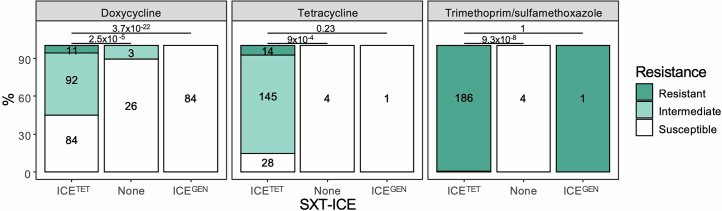
SXT-ICE elements and phenotypic antibiotic resistance. Proportion of samples that were phenotypically resistant to Trimethoprim/sulfamethoxazole, tetracycline and doxycycline using commercially available antibiotic discs, categorised by SXT-ICE element. The number of samples falling within each category is indicated on the bar chart (a total of n = 192 independent samples for Trimethoprim/sulfamethoxazole and tetracycline, and n = 300 for doxycycline). P values for fisher tests are indicated above the chart.

Finally, as may be expected, phenotypic antibiotic susceptibility testing suggested that the loss of ICE^TET^ was associated with increased susceptibility to trimethoprim/sulfamethoxazole (*P* = 9.3 × 10^−8^; Fisher’s test; Extended Data Fig. 9), tetracycline (*P* = 9 × 10^−4^) and doxycycline (*P* = 2.5 × 10^−5^), whereas sBD1 lineage *V. cholerae* carrying the ICE^GEN^ element was significantly more susceptible to doxycycline (*P* = 3.7 × 10^−22^).

## Discussion

The Sundarbans and highly populated areas of the Ganges Delta region in Bangladesh, bordering the Bay of Bengal, are considered by many as the heartland of epidemic cholera^2^. This notion is reinforced by genomic studies showing that a discrete clone of *V. cholerae*, named 7PET, radiated from this region in several epidemic waves to cause the continuing seventh pandemic^1^. 7PET is responsible for the current increase in cholera cases globally and the reason that the World Health Organization has declared cholera resurgence as a grade 3 emergency^29^. Although the global spread of cholera is evident, with 667,000 cholera cases and five of six World Health Organization regions reporting outbreaks in 2023 (ref. ^30^), little is known about the evolutionary dynamics of *V. cholerae* at its global source. Here we used genomics to understand the dynamics of *V. cholerae* across the greater Ganges region, including both the Delta regions within Bangladesh and India as well as the upper Ganges Basin covering eight North Indian states. We followed the evolution of sBD1 and BD2, the dominant circulating 7PET lineages in this region during this period.

Our results show that despite the Ganges Delta and Basin spanning Bangladesh and India, there is an apparent tendency for cholera evolution to follow national borders rather than hydrological features and flow. Both Kolkata and Assam harboured cholera sublineages present in North India but not in Bangladesh, whereas in Bangladesh the evolution of BD2 occurred almost in isolation from the rest of the world. Furthermore, many of the genetic changes in BD2 seemed to spread nationwide from Dhaka, where population density is greatest. Although there may be bias, with under-representation of samples from East India, this adds to the overwhelming evidence that 7PET cholera transmission is primarily mediated by short-cycle human-to-human transmission.

*V. cholerae* evolution in Bangladesh was characterized by rapid changes in genes and MGEs, particularly those relating to phage defence. Sublineages harbouring these changes rapidly became dominant, suggesting that *V. cholerae* evolution is driven by strong selective pressures. This is illustrated by the almost simultaneous switch in *V. cholerae* phage defence systems and ICP1 anti-defence systems in 2018. It is possible that once ICP1 co-evolved to overcome phage defences in BD2, retaining these systems no longer presented an evolutionary advantage, resulting in their sequential loss. PLE1 was independently lost at several points in the evolution of BD2, signifying that its maintenance presented a strong burden. A similar pattern was observed for the anti-phage *ddmABC* operon; once a frameshift mutation in *ddmC* rendered the operon ineffective^31^, the rest of the operon was deleted. We found that degradation of the *ddmABC* operon was associated with reduced prevalence of lysogenic phage K139. Although a causative mechanism is unclear from the data presented here, ongoing research on the structure and function of the complex may lead to a potential explanation^32–34^.

Having lost its defences, the BD2 lineage was then rapidly replaced by sBD1, harbouring an ICE^GEN^ element containing an anti-phage BREX system^10^ that was probably initially effective against local ICP1. However, ICP1 with an *odn* anti-defence system, possibly introduced alongside sBD1 from India, then rapidly became dominant. Finally, sBD1 overcomes this through acquisition of anti-phage island PLE11, which prevents ICP1 propagation by disrupting tail assembly, therefore acting as a strong evolutionary driver for ICP1 evolution^35^. Further, our data revealed that carriage of these phage defence systems was associated with lower disease severity and *V. cholerae* load, suggesting that their maintenance imposes a significant cost to pathogenicity. However, as our analysis of disease severity was limited to samples from Bangladesh, it remains unclear whether the same pattern holds elsewhere. The loss of ICE^TET^, which confers tetracycline resistance, indicates that antibiotic treatment does not exert as strong an evolutionary pressure.

Although PLE anti-phage islands were rare outside Bangladesh, both BD2 and sBD1 gained a PLE approximately 2 years after becoming prevalent in Bangladesh. Furthermore, PLEs were under-represented among transmission events out of Bangladesh, suggesting they may be advantageous only within the Ganges Delta, and compromise long-range transmission both within and beyond South Asia. This may partially explain why the inferred source of most global transmission events seems to be India rather than Bangladesh, refining the previous notion that the Ganges Delta holds the global diversity of all epidemic 7PET *V. cholerae* sublineages. Rather, surrounding countries, including India, serve as the launchpads for global transmission.

At present, it is unclear why PLEs seem to be largely confined to Bangladesh, as ICP1 has also been found in India, Yemen and the Democratic Republic of Congo^6,36,37^. Our hypothesis is that Bangladesh is one of the few places with a sufficiently high human population density and cholera prevalence, combined with high exposure rates stemming from a lack of preventative water, sanitation and hygiene (WASH) infrastructure, to enable effective transmission within this population while maintaining costly anti-phage defence systems. Furthermore, we found that Inaba serotype *V. cholerae* rarely transmitted outside Bangladesh, suggesting that although serotype switching may confer immune evasion in populations with high Ogawa-specific seroprevalence^38,39^, it could also impose a fitness cost in immune-naive populations.

In summary, we found that *V. cholerae* evolution in Bangladesh was unique in its rapid gain and loss of MGEs. This followed relatively predictable patterns, gaining PLEs when becoming established in Bangladesh and losing them over time, probably driven by the trade-off between maintaining costly anti-phage defences against ICP1 and pathogenicity—classical gene-for-gene theory but here on a regional scale^22,40,41^. Furthermore, in Bangladesh, *V. cholerae* seemed to radiate from areas of high population density, such as Dhaka and Chittagong. Real-time longitudinal genomic surveillance of *V. cholerae* in these regions could be used as an early-warning system for the entire country, identifying emerging sublineages harbouring changes predicted to increase risk of severe disease or antibiotic resistance. This could allow rapid mobilization of intervention strategies, such as vaccination or WASH, to prevent dissemination of high-risk sublineages. Given the rising threat of antibiotic resistance, phage therapy is of significant interest now as a new intervention strategy. Although our results should be considered in relation to using ICP1 as a therapeutic agent, the capacity for co-evolution may differ for other phages.

We also found that MGEs, such as PLEs, could compromise the global transmission potential and export of *V. cholerae* from Bangladesh to the upper Ganges region. Thus, despite the high prevalence and diversity of cholera in Bangladesh, the Ganges Delta may not represent the modern global source of cholera but rather India and the Ganges Basin as a whole. From our data, it is clear that focusing cholera surveillance on outbreaks in Africa, the Middle East and Haiti alone is insufficient to end the seventh cholera pandemic. Global annual cholera surveillance, akin to the surveillance and response system in place for influenza at present, will be necessary to effectively target sources of transmission using WASH interventions and limited oral cholera vaccine stockpiles.

## Methods

### Ethics statement

Study procedures for the Bangladesh 2014–2018 surveillance study and 2% icddr,b Dhaka Hospital Surveillance study were approved by the Research Review Committee and Ethical Review Committee of icddr,b. Participants provided informed written consent for data and sample collection. The study conducted in North India was approved by the Institute Ethics Committee and the Institute Collaborative Committee of PGIMER, Chandigarh. Written informed consent was obtained from all participants before data and sample collection.

### Sample collection and sequencing in Bangladesh

Study procedures for the 2014–2018 surveillance study were outlined in detail in ref. ^42^ and approved by the Research Review Committee and Ethical Review Committee of icddr,b. Sentinel surveillance was carried out at ten sites from 2014 to 2016, interrupted from January to May 2016 because of a gap in funding and subsequently expanded to 22 sites in 21 districts from May 2016 onwards. Informed written consent was obtained from all adult participants or from legal guardians for children younger than 18 years old. A stool sample was collected for detection of *V. cholerae* O1/O139. At the ten sites where surveillance was established in 2014, samples were also tested for enterotoxigenic *Escherichia coli*, *Salmonella* and *Shigella* species. Among 26,221 patients with acute watery diarrhoea, 6.2% (*n* = 1,604) were confirmed as* V. cholerae* O1 cases, of which 1,526 underwent whole-genome sequencing (Supplementary Fig. 7). Similarly, from the 2% icddr,b Dhaka Hospital Surveillance, in which every 50th patient visiting the icddr,b Hospital in Dhaka was enrolled and tested for enteric pathogens, 63 *V. cholerae* samples collected from 2022 to 2023 were also sequenced.

From the sentinel sites, stool samples were transported in Cary–Blair medium to icddr,b within 15 days of collection^42^. Samples were streaked directly onto taurocholate–tellurite gelatin agar and enriched in alkaline peptone water (pH 8.6) for 18 h before plating on taurocholate–tellurite gelatin agar. These were incubated overnight at 37 °C. Suspected *V. cholerae* colonies were serotyped using O1 Ogawa-specific, O1 Inaba-specific and O139-specific antibodies. A subsample (every fifth *V. cholerae*-positive culture) underwent antibiotic susceptibility testing (doxycycline, *n* = 311; tetracycline, *n* = 195; erythromycin, *n* = 366; azithromycin, *n* = 350; ciprofloxacin, *n* = 350; trimethoprim/sulfamethoxazole; *n* = 195; nalidixic acid, *n* = 140) using commercially available antibiotic discs (Oxoid) following the guidelines of the Clinical and Laboratory Standards Institute^43^. In 2017, testing for ampicillin (*n* = 187), ceftriaxone (*n* = 171) and cefixime (*n* = 171) was introduced. *E. coli* American Type Culture Collection 25922 susceptible to all antimicrobials was used as a control strain.

Genomic DNA was extracted from 5-ml cultures of *V. cholerae* incubated overnight at 37 °C in Luria–Bertani medium using the Wizard Genomic DNA Kit (Promega). DNA integrity was confirmed by agarose gel electrophoresis, and purity was evaluated with a NanoDrop 2000 spectrophotometer (Thermo Fisher Scientific). For the 2014–2018 surveillance study, 150-bp paired-end sequencing was carried out at the Wellcome Sanger Institute using the HiSeq 2500 platform (Illumina). Read data have been deposited in the European Nucleotide Archive (ENA) database under study accession ERP112767. For the 2% icddr,b Dhaka Hospital Surveillance study, paired-end sequencing was carried out using NextSeq 2000. Read data have been deposited in the ENA database under study accession ERP167534. FASTQ files were trimmed using fastp v.0.23.4, moving a sliding window from the 5′ and 3′ ends of the reads and trimming bases with a mean quality below 20. Read quality was verified using FastQC v.0.11.8 and MultiQC v.1.8.

### Sample collection and sequencing in North India

This study was approved by the Institute Ethics Committee of PGIMER, Chandigarh. Stool and water samples were collected during an outbreak investigation in affected areas of Chandigarh and neighbouring states in North India. Additionally, water samples were obtained from freshwater sites, including rivers and ponds. The stool and water samples were transported in Cary–Blair medium and under a cold chain, respectively, to PGIMER, Chandigarh, for processing.

Water samples were filtered using 0.22-μm nitrocellulose acetate filters, followed by vortexing in phosphate-buffered saline to release adherent cells. Both filtered water samples (100-μl aliquots) and stool samples were enriched in alkaline peptone water at 37 °C for 6–8 h. This was followed by subculturing the enriched samples onto blood agar and thiosulfate–citrate–bile salts–sucrose agar for further incubation at 37 °C for 18–24 h. The collected samples were also tested for other enteric pathogens, such as *Shigella* and *Salmonella*. Colonies resembling *V. cholerae* or other enteric pathogens were identified using matrix-assisted laser desorption/ionization–time of flight. Genomic DNA was extracted as described above. High-throughput genome sequencing was carried out on the Illumina platform to generate 150-bp paired-end reads, and quality control of the sequencing data was performed as described above. Read data have been deposited in the ENA database under study accessions ERP188886 and ERP188887.

### Phylogenetic analysis

We contextualized these resulting genomes within a global collection of 7PET genomic sequences (Supplementary Data 1). Reads were mapped against *V. cholerae* N16961 using snippy v.4.6 (ref. ^44^) to create a pseudogenome alignment. Snp-sites v.2.5.1 (ref. ^45^) was used to create an SNP-only alignment, and snp-dists v.0.7 (ref. ^46^) was used to calculate pairwise distances between genomes. Kraken v.1.1.1 (ref. ^47^) was used to find the proportion of reads attributed to *V. cholerae* and ICP1. Samples with an SNP distance greater than 400 from reference strain N16961 or less than 90% reads attributed to *V. cholerae* were excluded. The 7PET samples were divided into major lineages using rhierBAPS^48^ and sublineages on the basis of a pairwise SNP distance smaller than 20. Maximum likelihood phylogenetic trees for (1) global 7PET and (2) sBD1 and (3) BD2 within the systematic 2014–2018 surveillance study were created using IQ-TREE v.1.6.12 using the HKY+F+I substitution model^49^. TreeTime v.0.7.4 (ref. ^50^) was used to re-root trees and create a maximum-likelihood time-scaled phylogeny. The discrete ancestral state of each node, including location, sublineage and the presence of key genes and MGEs, was estimated using TreeTime ‘mugration’. Transitions between ancestral locations in the global 7PET tree were used to estimate transmission between countries, whereas transitions between locations in the sBD1 and BD2 systematic 2014–2018 surveillance study trees were used to infer transmission events between divisions within Bangladesh. Phylogenetic trees were visualized using the R package APE v.5.8 (ref. ^51^) and ggtree v.3.12.0 (ref. ^52^). Maps were generated in R (sf package v.1.0-21; ref. ^53^) using publicly available spatial vector datasets, including Natural Earth (public domain; R package rnaturalearth v.1.1.0; ref. ^54^), Bangladesh R package v.1.0.0 (ref. ^55^), HydroRIVERS (HydroSHEDS database^56^) and World Bank Major River Basins dataset^57^.

### Subsampling

We used repeated subsampling to verify whether sampling intensity affected inferred transmission events between regions and countries. For interregional transmission events inferred from the 2014–2018 Bangladesh nationwide surveillance study, we carried out repeated subsampling (ten repeats) to give the ratios we would expect if the proportion of acute watery diarrhoea cases from each region each year reflected the proportion of the Bangladesh population living in that region. We used the maximum number of samples possible to achieve this ratio each year. Mymensingh was excluded, and Rajshahi/Rangpur and Khulna/Barisal were grouped into Northern Bengal and Southern Bengal, respectively, to increase the number of samples within each region. For each subsample, we constructed phylogenetic trees for sBD1 and sBD2 using IQ-TREE v.1.6.12. TreeTime v.0.7.4 was used to re-root trees and infer the ancestral state of each node.

For international transmission events, samples from the entire collection of *V. cholerae* genomes were subsampled (ten repeats) such that the numbers of samples from Bangladesh, India and the rest of the world were equal each year. We used the maximum number of samples possible to achieve this each year. For each subsample, we constructed a 7PET phylogenetic tree using IQ-TREE v.1.6.12. As described above, TreeTime v.0.7.4 was used to re-root trees and infer the ancestral state of each node.

As nucleotide diversity (π) is affected by the number of genomes analysed, to compare nucleotide diversity in Bangladesh and India over time, repeated subsamples (ten repeats) of 15 samples per country per year were taken. Pegas v.1.3 (ref. ^58^) was used to calculate nucleotide diversity and Tajima’s *D* for each subsample each year.

### Virulence genes, antibiotic resistance and mobile genetic elements

*V. cholerae* genome assembly was carried out using SPAdes v.4.1.0. ARIBA v.2.14.6 (ref. ^59^) was used to detect the presence of complete reading frames for antibiotic resistance genes (against the Comprehensive Antibiotic Resistance Database), virulence factors (against the Virulence Factor Database), *ctxB* types, all genes present in the N16961 reference genome and ICP1 anti-defence genes (reference genome MW794190.1 for *csy1-4*, *cas1* and *cas3* genes; MW794192.1 for the *odn* gene). Mash v.2.1.1 (ref. ^60^) was used to screen for sample read sets contained within MGE sequences, including PLE1 (KC152960.1), PLE2 (KC152961.1), PLEs 3−10 (ref. ^41^), PLE11 (identified from de novo genome assemblies using Panaroo v.1.3.4; ref. ^61^), SXT-ICE^TET^ (MK165649.1), SXT-ICE^GEN^/ICEVchInd5 (KY382507.1), ICEVchBan9 (CP001485), ICEVchCHN143 (KT151654), ICEVchInd4 (GQ463141) and plasmids pCNRVC190243 (ref. ^6^) (OW443149.1) and pSAG71 (CP053818.1). An MGE was considered present if a sample shared 700 of 1,000 hashes. Samples co-sequenced with ICP1 were identified on the basis of greater than 0.1% of reads mapping to ICP1 using Kraken v.1.1.1. The association of key genes and MGEs with rice-water stool, severe dehydration and K139 prevalence was assessed using a multivariate logistic regression model implemented in R, adjusting for the presence of all other key genes/MGEs, time and sampling site.

### Secondary metagenomic analysis

We carried out a systematic screen of metagenomes from 40 cholera-endemic countries (Supplementary Table 1). National Center for Biotechnology Information Sequence Read Archivewas searched for samples that underwent Illumina-based whole-genome DNA sequencing and fell within taxonomy IDs 408170 (human gut metagenome), 2705415 (human faeces metagenome) or 749906 (gut metagenome) with *Homo sapiens* listed as the host. Sylph v.0.8.1 (ref. ^62^) was used to rapidly screen for the presence of *V. cholerae* using a custom database containing reference genome N16961. Kraken2 v.2.0.8 was used to screen for ICP1 using a database containing only RefSeq viruses. Mash v.2.1.1 was used to detect MGEs. Samples with more than 1,000 reads mapping to ICP1 were assembled using SPAdes v.4.1.0, and contigs corresponding to ICP1 were identified using BLAST v.2.7.1 against reference GCA_000893175.1. We also downloaded 60 ICP1 assemblies from NCBI and identified ICP1 contigs from 74 assemblies, in which ICP1 was co-sequenced alongside *V. cholerae*. Assemblies with more than 50% coverage of reference GCA_000893175.1 were annotated using Prokka v.1.14.5, and a core gene alignment was generated using panaroo v.1.3.4. Snp-sites v.2.5.1 was used to create an SNP-only alignment, and IQ-TREE v.1.6.12 (HKY+F+I substitution model) was used to build a phylogenetic tree. The tree was midpoint rooted using phytools v.2.4-4 (ref. ^63^). BLAST v.2.7.1 was used to identify the presence of specific genes within assemblies.

### Reporting summary

Further information on research design is available in the Nature Portfolio Reporting Summary linked to this article.

## Online content

Any methods, additional references, Nature Portfolio reporting summaries, source data, extended data, supplementary information, acknowledgements, peer review information; details of author contributions and competing interests; and statements of data and code availability are available at 10.1038/s41586-026-10340-x.

## Supplementary information

Supplementary Information Supplementary Figs. 1–7 and Supplementary Table 1.

Reporting Summary

Supplementary Data 1 Global collection of 7PET *V. cholerae* genomes.

Supplementary Data 2 Inferred ancestral states of nodes in the 7PET *V. cholerae* tree.

Supplementary Data 3 Gut metagenomes from cholera-endemic countries.

Supplementary Data 4 Global collection of ICP1 genomes.

## Data Availability

The read data generated in this study have been deposited in the ENA database under study accessions ERP112767 (2014–2018 Bangladesh surveillance), ERP167534 (2% icddr,b Dhaka Hospital Surveillance study) and ERP188886 and ERP188887 (North India samples). Individual sample accessions for newly generated and publicly available data are indicated in Supplementary Data 1. Data to reproduce the figures and analyses are available at Zenodo (10.5281/zenodo.18786011)^64^.

## Code availability

Code to reproduce the figures and analyses are available at Zenodo (10.5281/zenodo.18786011)^64^.
