# Supplementary material for: Evolution of pandemic cholera at its global source
Source: Nature. 2026 Apr 1;653(8114):491–8. doi: 10.1038/s41586-026-10340-x (PMC13171446; doi:10.1038/s41586-026-10340-x)
Supplement: Supplementary file 1 — Supplementary Figs. 1–7 and Supplementary Table 1. [file 41586_2026_10340_MOESM1_ESM.docx]

**Evolution of Pandemic Cholera at its Global Source: Supplementary information**

Amber Barton*^†1^, Mokibul Hassan Afrad^†2^, Alyce Taylor-Brown^1^, Nisha Singh^1,3^, Chetan Thakur^1,3^, Taufiqul Islam^2^, Sadia Isfat Ara Rahman^2^, Marjahan Akhtar^2^, Yasmin Ara Begum^2^, Taufiqur Rahman Bhuiyan^2^, Ashraful Islam Khan^2^, Neelam Taneja^3^, Nicholas R. Thomson*^$1,4^, Firdausi Qadri*^$2^

*Corresponding authors: [ab61@sanger.ac.uk](mailto:ab61@sanger.ac.uk), [nrt@sanger.ac.uk](mailto:nrt@sanger.ac.uk), [fqadri@icddrb.org](mailto:fqadri@icddrb.org)

^†^Contributed equally

^$^Contributed equally

1. Parasites and Microbes, Wellcome Sanger Institute, Wellcome Genome Campus, Hinxton, Cambridgeshire, UK

2. Infectious Diseases Division, International Centre for Diarrhoeal Disease Research, Bangladesh (icddr,b), Dhaka, Bangladesh

3. Department of Medical Microbiology, Postgraduate Institute of Medical Education & Research, Chandigarh, 160012, India

4. Department of Pathogen Molecular Biology, Faculty of Infectious and Tropical Diseases, London School of Hygiene & Tropical Medicine, London

**_________________________________________________________________________________**

**Contents**

Supplementary Figure 1: Samples included in each analysis………………………………………..**Page 2**

Supplementary Figure 2: Time-scaled reference-mapped SNP phylogeny of sBD1 and BD2…..**Page 3**

Supplementary Figure 3: Inferred inter-regional transmission events following sub-sampling to account for regional variation in sampling of acute watery diarrhoea cases…………………………………**Page 4**

Supplementary Figure 4: Nucleotide diversity and Tajima’s D in Bangladesh and India each year, based on repeated sub-samples of 15 samples per country per year………………………………………**Page 5**

Supplementary Figure 5: Dynamics of PLEs, *ddmABC* (VC0492-490) and *wbeT* (VC0258) in different countries and regions worldwide………………………………………………………………………..**Page 6**

Supplementary Figure 6: Inferred international transmission events by source country following sub-sampling to account for differences in sampling intensity each year ………………………………**Page 7**

Supplementary Figure 7: Proportion of the population, acute watery diarrhoea cases, cholera cases, sequenced samples, and samples included in the final analysis, from each administrative division of Bangladesh in the 2014-2018 systematic surveillance study……………………………………….**Page 8**

Supplementary Table 1: Number of human gut metagenomes identified in a systematic search of NCBI SRA…………………………………….………………………………………………………………….**Page 9**


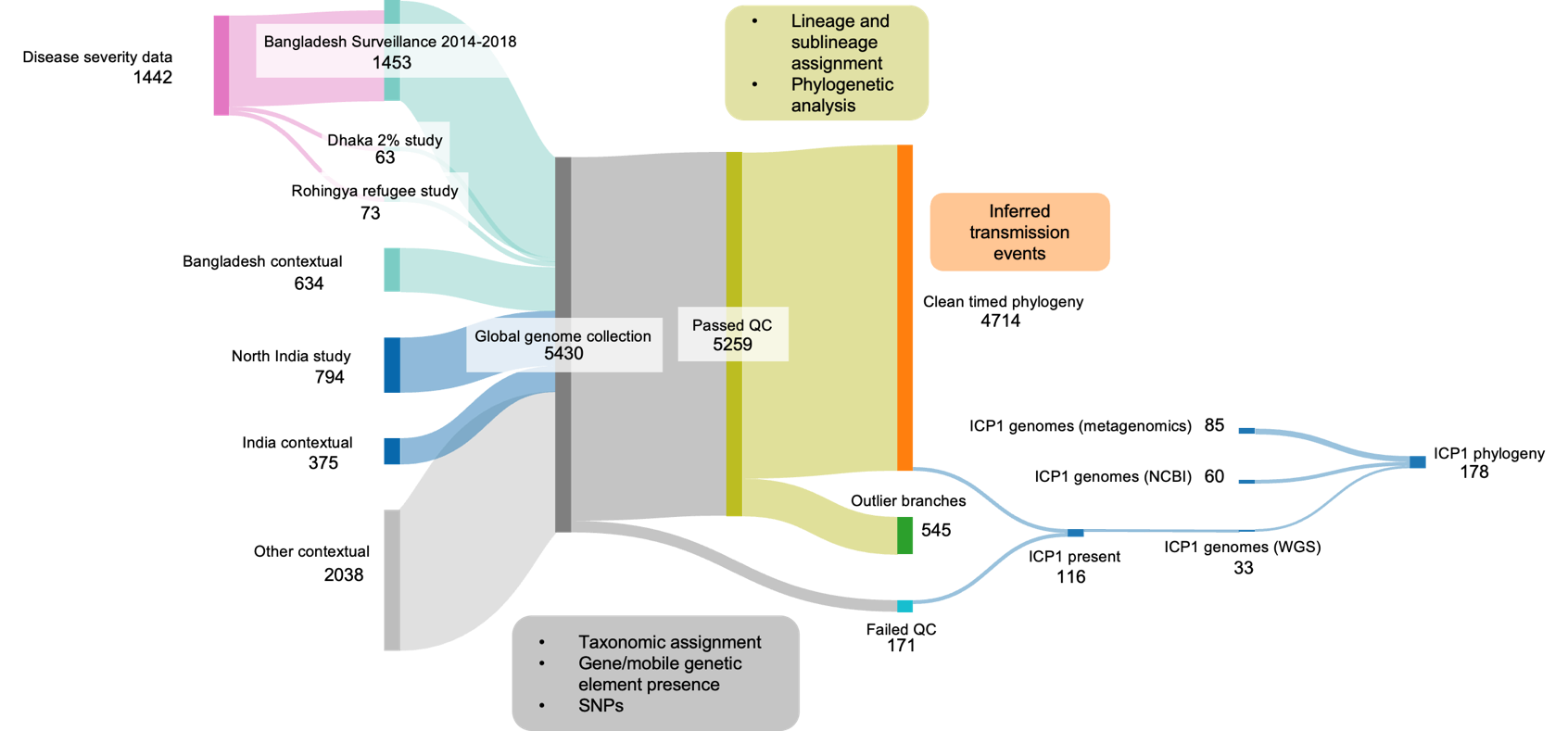


Supplementary Figure 1: Schematic of the number of samples included in each analysis. Diagram created using SankeyMATIC


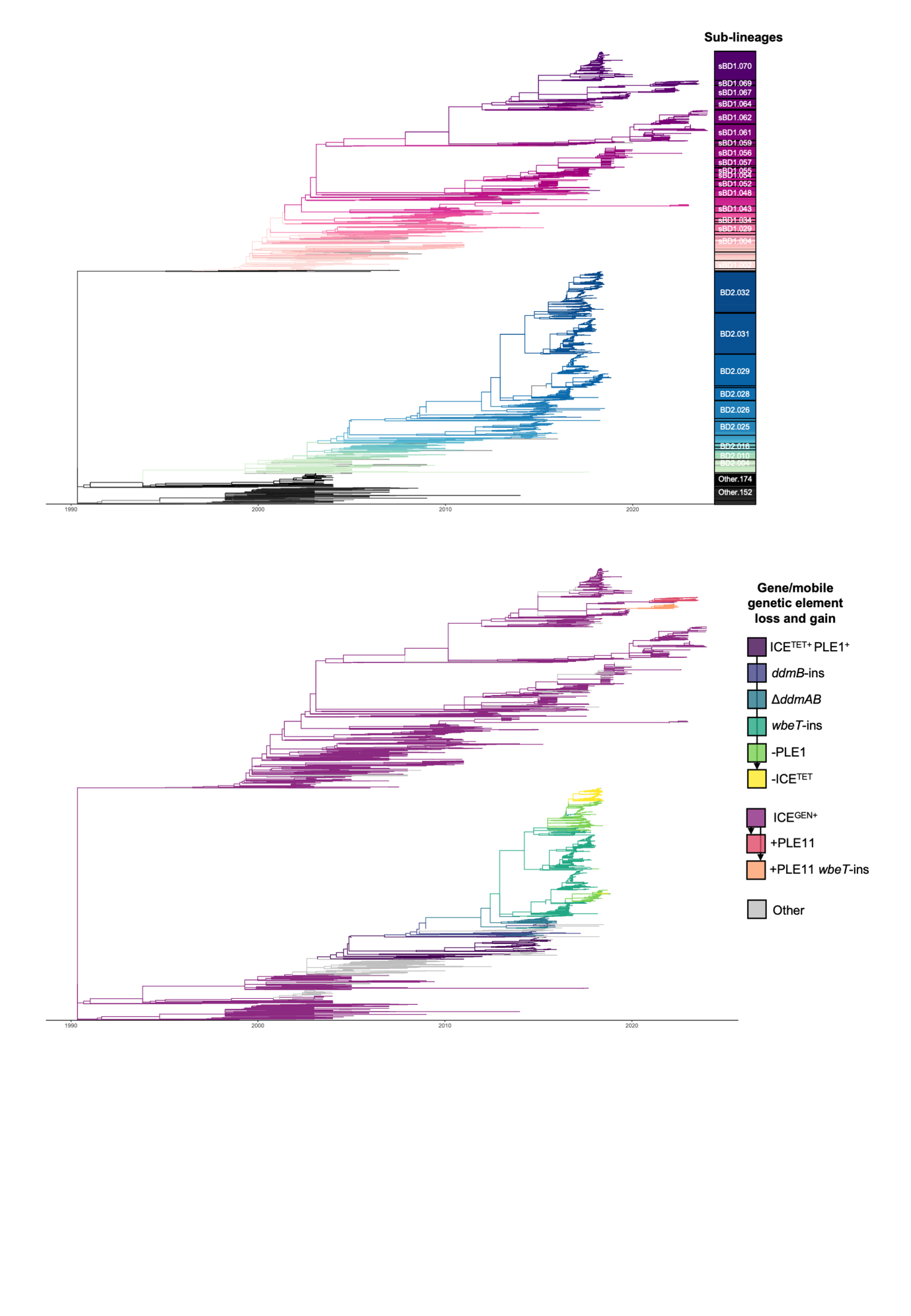


Supplementary Figure 2: Time-scaled reference-mapped SNP phylogeny of sBD1 and BD2. Outlier branches that did not follow a molecular clock have been excluded. Branches are coloured by sub-lineage or gene/mobile genetic element profile


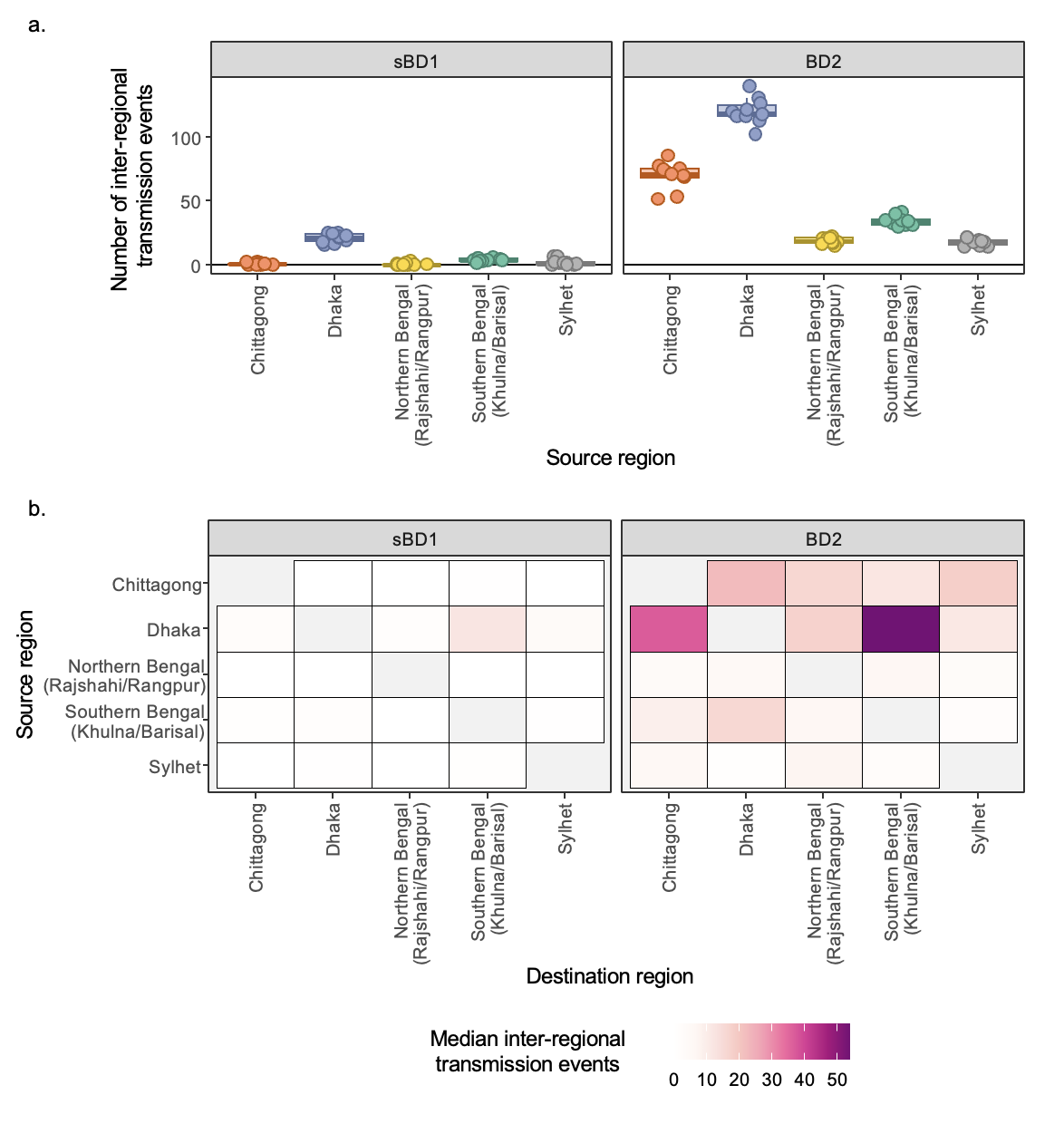


Supplementary Figure 3: Inferred inter-regional transmission events following sub-sampling to account for regional variation in sampling of acute watery diarrhoea cases. a. Number of inferred transmission events from each region, sub-categorised by lineage. Each point represents one sub-sample. A total of 10 sub-samples were carried out for each lineage. b. Median number of inferred transmission events between each pair of regions, sub-categorised by lineage.


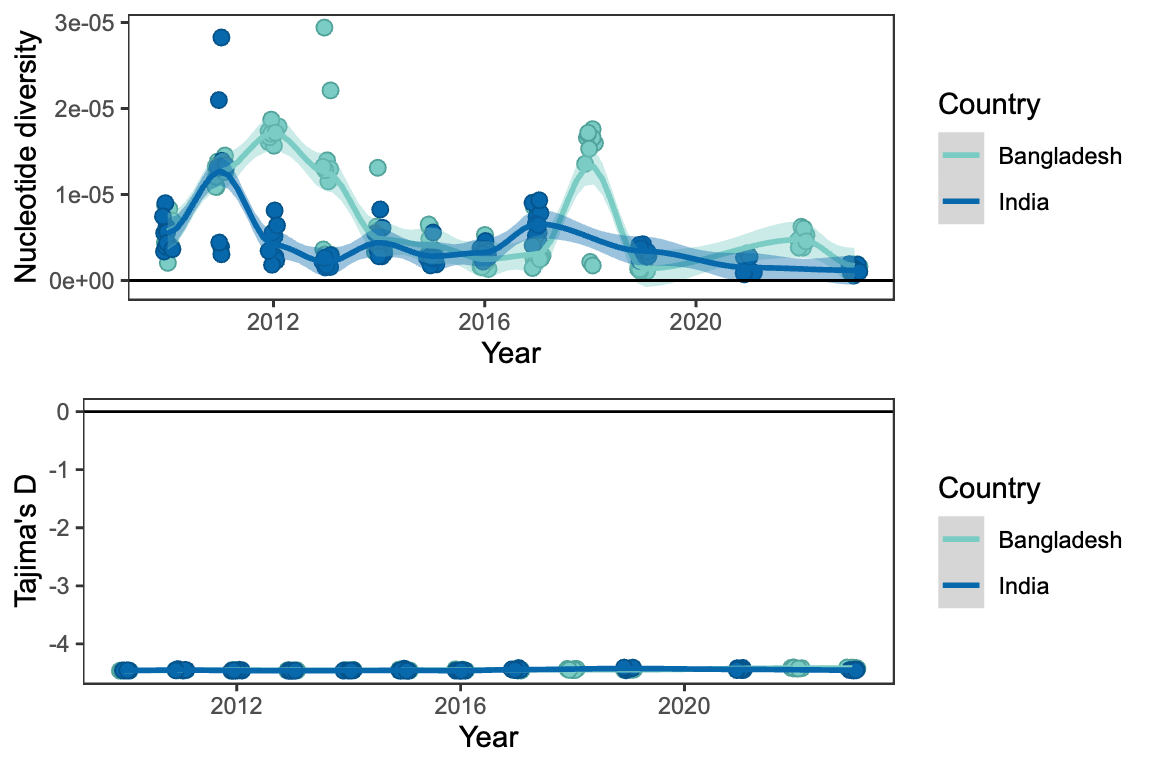


Supplementary Figure 4: Nucleotide diversity and Tajima’s D in Bangladesh and India each year, based on repeated sub-samples of 15 samples per country per year.


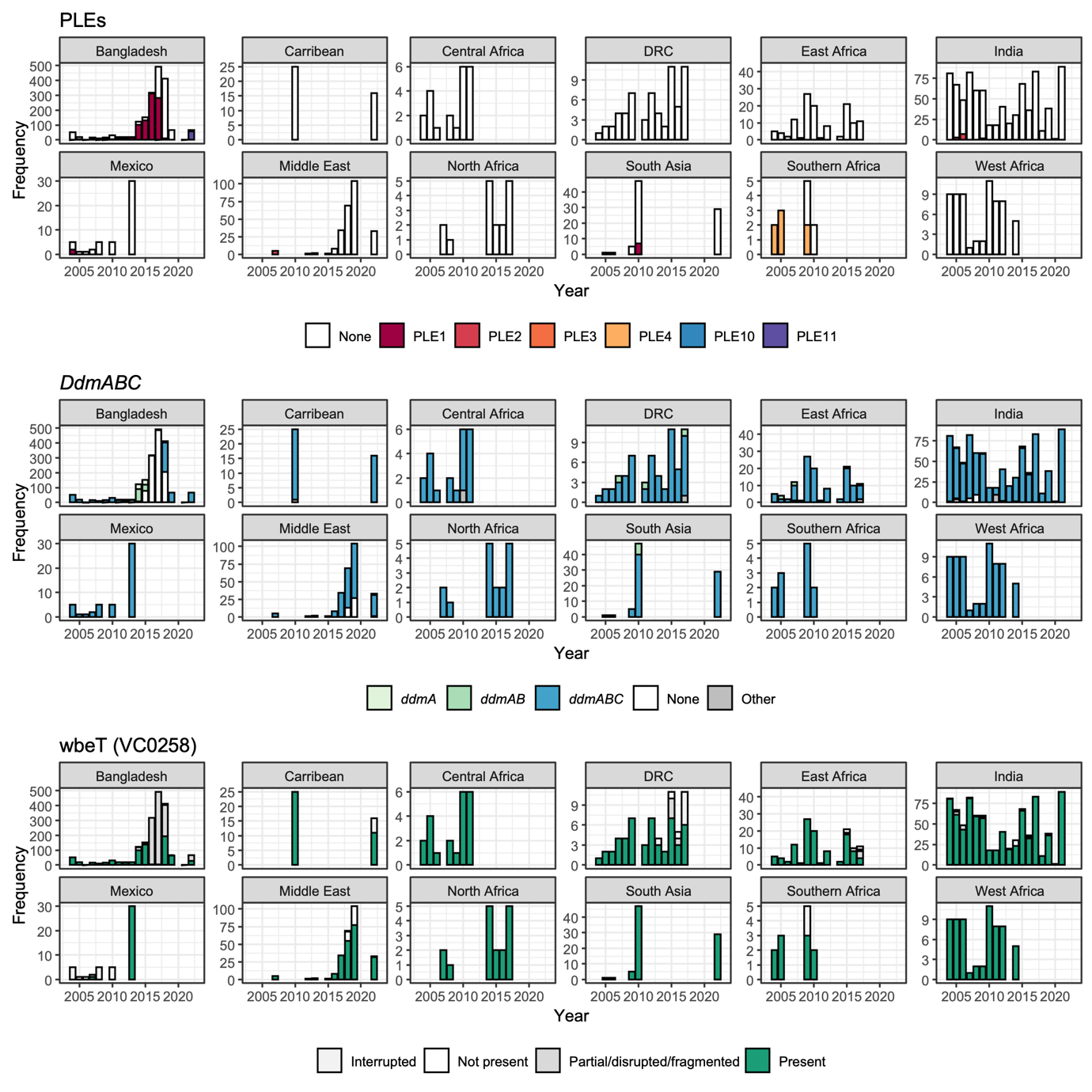


Supplementary Figure 5: Dynamics of PLEs, *ddmABC* (VC0492-490) and *wbeT* (VC0258) in different countries and regions worldwide. Bars are coloured according to the frequency of PLE or gene presence in each country/region each year from 2003-2023. n=4674 samples.


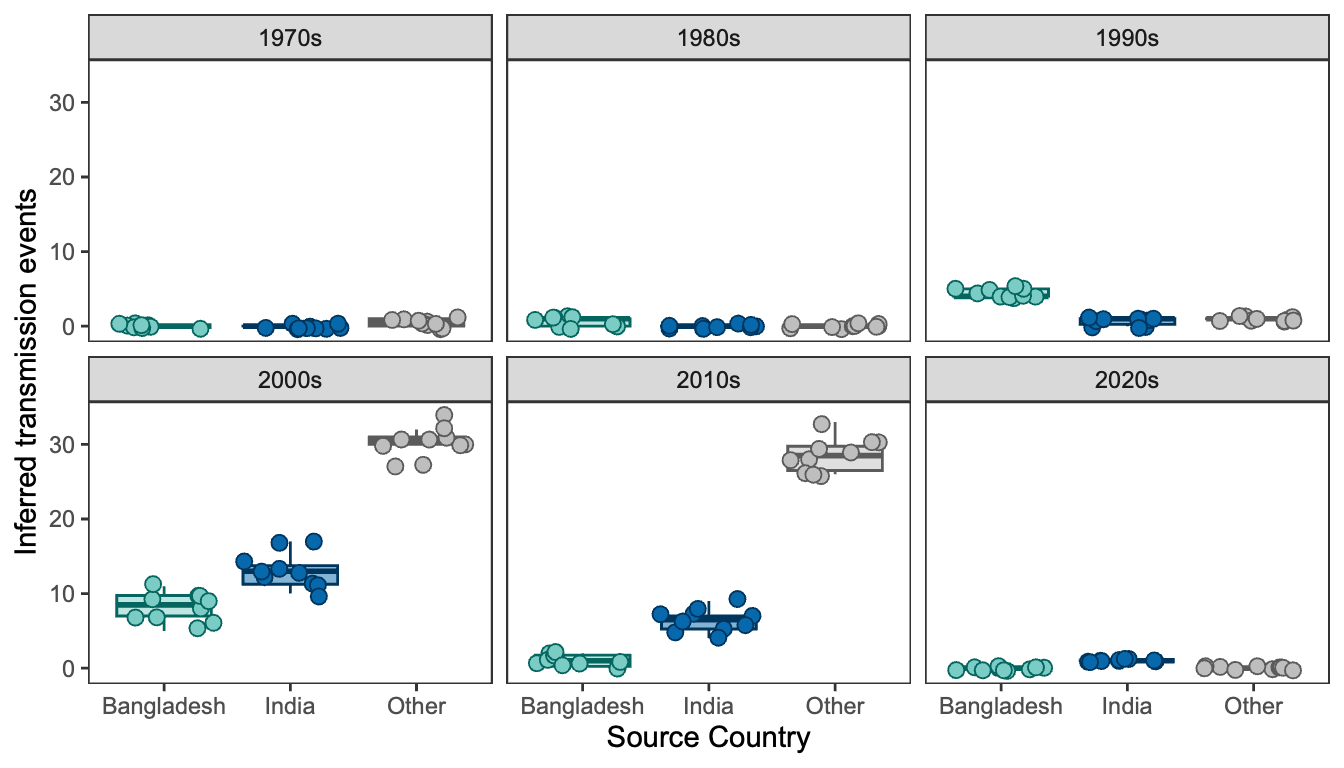


Supplementary Figure 6: Inferred international transmission events by source country following sub-sampling to account for differences in sampling intensity each year. Each point represents one sub-sample. A total of 10 sub-samples were carried out.


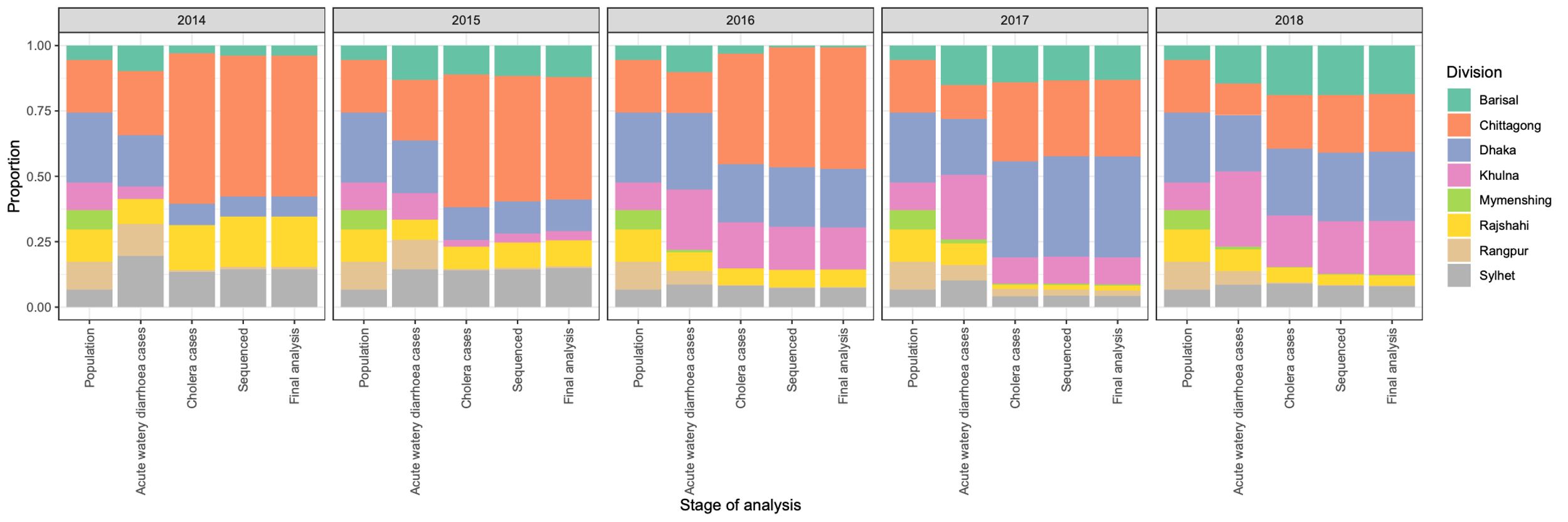


Supplementary Figure 7: Proportion of the population, acute watery diarrhoea cases, cholera cases, sequenced samples, and samples included in the final analysis, from each administrative division of Bangladesh in the 2014-2018 systematic surveillance study.

Supplementary Table 1: Number of human gut metagenomes identified in a systematic search of NCBI SRA

| **Country** | **Metagenomes** |
| --- | --- |
| Afghanistan | 0 |
| Angola | 0 |
| Bangladesh | 4487 |
| Benin | 0 |
| Bhutan | 0 |
| Burkina Faso | 1210 |
| Burundi | 0 |
| Cameroon | 237 |
| China | 0 |
| Democratic Republic of the Congo | 180 |
| Dominican Republic | 0 |
| Ethiopia | 87 |
| Ghana | 525 |
| Haiti | 492 |
| India | 1275 |
| Iran | 40 |
| Iraq | 0 |
| Kenya | 598 |
| Lebanon | 0 |
| Malawi | 124 |
| Mozambique | 263 |
| Myanmar | 0 |
| Namibia | 0 |
| Nepal | 57 |
| Niger | 434 |
| Nigeria | 27 |
| Pakistan | 13 |
| Philippines | 85 |
| Rwanda | 0 |
| Somalia | 0 |
| South Sudan | 0 |
| Sudan | 0 |
| Syria | 0 |
| Tanzania | 488 |
| Thailand | 381 |
| Togo | 0 |
| Uganda | 236 |
| Yemen | 0 |
| Zambia | 0 |
| Zimbabwe | 1866 |
