# Supplementary material for: Evolution of pandemic cholera at its global source
Source: Nature. 2026 Apr 1;653(8114):491–8. doi: 10.1038/s41586-026-10340-x (PMC13171446; doi:10.1038/s41586-026-10340-x)
Supplement: Supplementary file 2 — Reporting Summary [file 41586_2026_10340_MOESM2_ESM.pdf]

nature portfolio

Corresponding author(s): Amber Barton
Last updated by author(s): 2/20/2026

Reporting Summary

Nature Portfolio wishes to improve the reproducibility of the work that we publish. This form provides structure for consistency and transparency in reporting. For further information on Nature Portfolio policies, see our Editorial Policies and the Editorial Policy Checklist.

Statistics

For all statistical analyses, confirm that the following items are present in the figure legend, table legend, main text, or Methods section.

n/a	Confirmed	
☐	☒	The exact sample size (*n*) for each experimental group/condition, given as a discrete number and unit of measurement
☒	☐	A statement on whether measurements were taken from distinct samples or whether the same sample was measured repeatedly
☐	☒	The statistical test(s) used AND whether they are one- or two-sided<br>*Only common tests should be described solely by name; describe more complex techniques in the Methods section.*
☐	☒	A description of all covariates tested
☒	☐	A description of any assumptions or corrections, such as tests of normality and adjustment for multiple comparisons
☐	☒	A full description of the statistical parameters including central tendency (e.g. means) or other basic estimates (e.g. regression coefficient) AND variation (e.g. standard deviation) or associated estimates of uncertainty (e.g. confidence intervals)
☐	☒	For null hypothesis testing, the test statistic (e.g. *F*, *t*, *r*) with confidence intervals, effect sizes, degrees of freedom and *P* value noted<br>*Give P values as exact values whenever suitable.*
☒	☐	For Bayesian analysis, information on the choice of priors and Markov chain Monte Carlo settings
☒	☐	For hierarchical and complex designs, identification of the appropriate level for tests and full reporting of outcomes
☒	☐	Estimates of effect sizes (e.g. Cohen's *d*, Pearson's *r*), indicating how they were calculated

Our web collection on statistics for biologists contains articles on many of the points above.

Software and code

Policy information about availability of computer code

Data collection	No software was used for data collection.
Data analysis	Phylogenetic analysis: snippy v4.6, Snp-sites v2.5.1, snp-dists v0.7<br>Quality control of whole genome sequencing: Kraken v1.1.1<br>Phylogenetic trees: IQ-tree v1.6.12 , TreeTime v0.7.4, ape v5.8, ggtree v3.12.0<br>Nucleotide diversity: Pegas v1.3<br>Vibrio Cholerae Assembly: spades v4.1.0<br>Annotation: PROKKA v1.14.5<br>Presence of genes and mobile genetic elements: ARIBA v2.14.6, Mash v2.1.1, kraken v1.1.1<br>Metagenome analysis: Sylph v0.8.1, kraken2 v2.0.8, mash v2.1.1, blast v2.7.1, PROKKA v1.14.5<br>ICP1 phylogeny: panaroo v1.3.4, Snp-sites v2.5.1, IQ-tree v1.6.12, phytools v2.4-4

For manuscripts utilizing custom algorithms or software that are central to the research but not yet described in published literature, software must be made available to editors and reviewers. We strongly encourage code deposition in a community repository (e.g. GitHub). See the Nature Portfolio guidelines for submitting code & software for further information.

Data

Policy information about availability of data

All manuscripts must include a data availability statement. This statement should provide the following information, where applicable:

- Accession codes, unique identifiers, or web links for publicly available datasets
- A description of any restrictions on data availability
- For clinical datasets or third party data, please ensure that the statement adheres to our policy

The read data generated in this study have been deposited in the ENA database (ebi.ac.uk/ena) under the study accessions ERP112767 (2014-2018 Bangladesh surveillance), ERP167534 (2% icddr,b Dhaka Hospital Surveillance study), ERP188886 and ERP188887 (North India samples). Individual sample accessions for newly generated and publicly available data are indicated in Supplementary Data 1.

Research involving human participants, their data, or biological material

Policy information about studies with human participants or human data. See also policy information about sex, gender (identity/presentation), and sexual orientation and race, ethnicity and racism.

Reporting on sex and gender	Data on sex was recorded for the cholera surveillance studies in Bangladesh (N = 709 males and 603 females for the 2014-2018 surveillance study, N = 37 males and 24 females for the 2% study). However sex-based analyses were not performed as the study focused on the biology and genetics of the bacterial pathogen and did not use any human material.
Reporting on race, ethnicity, or other socially relevant groupings	This study does not include reporting on race, ethnicity or other socially relevant groupings.
Population characteristics	Data on age was recorded for the cholera surveillance studies in Bangladesh (interquartile range 16-40 years for the 2014-2018 surveillance study, and 9.5-40 years for the 2% study). However age-based analyses were not performed as the study focused on the biology and genetics of the bacterial pathogen and did not use any human material.
Recruitment	2014-2018 Bangladesh surveillance study: Recruitment procedures are outlined in detail in Khan et al. 2020. Briefly, each day Saturday-Wednesday at each site, four patients with diarrhoea who met the case definition and had no other severe comorbidity were enrolled.<br>2% study: Every 50th patient visiting the icddr,b Hospital in Dhaka was enrolled and tested for enteric pathogens.<br>North India study: Samples were collected during an outbreak investigation in affected areas within Chandigarh and neighbouring states in North India.
Ethics oversight	Bangladesh studies: Research Review Committee and Ethical Review Committee of icddr,b<br>North India study: Institute Ethics Committee, PGIMER-Chandigarh

Note that full information on the approval of the study protocol must also be provided in the manuscript.

Field-specific reporting

Please select the one below that is the best fit for your research. If you are not sure, read the appropriate sections before making your selection.

☒ Life sciences ☐ Behavioural & social sciences ☐ Ecological, evolutionary & environmental sciences

For a reference copy of the document with all sections, see nature.com/documents/nr-reporting-summary-flat.pdf

Life sciences study design

All studies must disclose on these points even when the disclosure is negative.

Sample size	No sample size calculations were conducted: sample size depended on the number of stool samples collected from recruited participants that were identified as being positive for Vibrio cholerae O1.
Data exclusions	Samples with a SNP distance >400 from reference N16961 or <90% reads attributed to V. cholerae were excluded in order to limit analyses to 7PET lineage V. cholerae. These thresholds were pre-established.
Replication	The population structure of Vibrio cholerae was consistent with publicly available contextual genomes collected in Bangladesh and India, as well as publicly available stool metagenomes collected in Bangladesh. As this is an observational study, no experimental replication was performed. Analyses are fully reproducible through the available code and publicly accessible sequencing data.
Randomization	No group allocation was performed.
Blinding	No group allocation was performed.

Reporting for specific materials, systems and methods

We require information from authors about some types of materials, experimental systems and methods used in many studies. Here, indicate whether each material, system or method listed is relevant to your study. If you are not sure if a list item applies to your research, read the appropriate section before selecting a response.

Materials & experimental systems

n/a	Involved in the study
☒	☐ Antibodies
☒	☐ Eukaryotic cell lines
☒	☐ Palaeontology and archaeology
☒	☐ Animals and other organisms
☒	☐ Clinical data
☒	☐ Dual use research of concern
☒	☐ Plants

Methods

n/a	Involved in the study
☒	☐ ChIP-seq
☒	☐ Flow cytometry
☒	☐ MRI-based neuroimaging

Plants

Seed stocks

Report on the source of all seed stocks or other plant material used. If applicable, state the seed stock centre and catalogue number. If plant specimens were collected from the field, describe the collection location, date and sampling procedures.

Novel plant genotypes

Describe the methods by which all novel plant genotypes were produced. This includes those generated by transgenic approaches, gene editing, chemical/radiation-based mutagenesis and hybridization. For transgenic lines, describe the transformation method, the number of independent lines analyzed and the generation upon which experiments were performed. For gene-edited lines, describe the editor used, the endogenous sequence targeted for editing, the targeting guide RNA sequence (if applicable) and how the editor was applied.

Authentication

Describe any authentication procedures for each seed stock used or novel genotype generated. Describe any experiments used to assess the effect of a mutation and, where applicable, how potential secondary effects (e.g. second site T-DNA insertions, mosiacism, off-target gene editing) were examined.
